# EMT circulating tumor cells detected by cell-surface vimentin are associated with prostate cancer progression

**DOI:** 10.18632/oncotarget.17632

**Published:** 2017-05-04

**Authors:** Arun Satelli, Izhar Batth, Zachary Brownlee, Abhishek Mitra, Shouhao Zhou, Hyangsoon Noh, Christina R. Rojas, Heming Li, Qing H. Meng, Shulin Li

**Affiliations:** ^1^ Department of Pediatrics, The University of Texas MD Anderson Cancer Center, Houston, Texas, USA; ^2^ Department of Biostatistics, The University of Texas MD Anderson Cancer Center, Houston, Texas, USA; ^3^ Department of Laboratory Medicine, The University of Texas MD Anderson Cancer Center, Houston, Texas, USA

**Keywords:** prostate cancer, epithelial mesenchymal transition, circulating tumor cells, vimentin, castration resistance

## Abstract

Recent advances in the field of circulating tumor cells (CTC) have shown promise in this liquid biopsy-based prognosis of patient outcome. However, not all of the circulating cells are tumor cells, as evidenced by a lack of tumor-specific markers. The current FDA standard for capturing CTCs (CellSearch) relies on an epithelial marker and cells captured via CellSearch cannot be considered to have undergone EMT. Therefore, it is difficult to ascertain the presence and relevance of any mesenchymal or EMT-like CTCs. To address this gap in technology, we recently discovered the utility of cell-surface vimentin (CSV) as a marker for detecting mesenchymal CTCs from sarcoma, breast, and colon cancer. Here we studied peripheral blood samples of 48 prostate cancer (PCA) patients including hormone sensitive and castration resistant sub-groups. Blood samples were analyzed for three different properties including our own CSV-based CTC enumeration (using 84-1 mAb against CSV), CellSearch-based epithelial CTC counts, and serum prostate-specific antigen (PSA) quantification. Our data demonstrated that in comparison with CellSearch, the CSV-based method had greater sensitivity and specificity. Further, we observed significantly greater numbers of CTCs in castration resistant patients as measured by our CSV method but not CellSearch. Our data suggests CSV-guided CTC enumeration may hold prognostic value and should be further validated as a possible measurement of PCA progression towards the deadly, androgen-independent form.

## INTRODUCTION

Prostate cancer (PCA) is the second leading cause of death of men in the United States [1]. In spite of these statistics, a valid and highly reliable means of predicting its progression towards androgen-independence has not yet been developed. Current diagnostic approaches rely predominately on prostate-specific antigen (PSA), though there has been debate as to the effectiveness of this measure. In a landmark study conducted by Andriole et. al. which included over 70,000 men, the authors found that mortality due to PCA was not significantly reduced by the incorporation of regular PSA screenings [2]. Recent research further indicates that PSA may not be effective in differentiating between low-risk and aggressive types of PCA [3]. Clearly, the identification and validation of biomarkers that can better diagnose PCA disease progression and assess its severity are critically needed.

Recently, the use of circulating tumor cells (CTCs) has been explored as an alternate tool for detecting and predicting overall survival in PCA patients [4]. CTC counts have been shown to correlate with patient prognosis in breast, prostate, and colorectal cancer [5–8]. As a major portion of the “liquid biopsy,” CTC enumeration has the potential to significantly improve diagnostics and therapeutics for several cancers. Many techniques for the isolation and identification of these cells have been developed, based on both their physical and biological properties [9]. Most notable is the U.S. Food and Drug Administration-approved CellSearch system, an EpCAM antibody-dependent technology that isolates and enumerates epithelial CTCs. Specifically regarding PCA, Goldkorn et. al. found that changes in CTC counts were more reliable than PSA levels in determining patient prognosis [4]. Though further validation by large-scale studies is necessary, these data provide support for further exploration of CTC counts as indicators of PCA progression.

However, the use of many CTC isolation technologies, including CellSearch, is centered on EpCAM, an epithelial surface marker that has limitations [10]. While these counts have proven clinically relevant, various studies suggest that cells must undergo EMT in order to enter circulation and reach distant sites [11]. This transition results in morphological changes that confer CTCs their remarkable motility and invasiveness, as well as their ability to escape detection by the immune system [12]. Since the CellSearch technology depends on the EpCAM marker for the isolation of CTCs, it may not detect the critical mesenchymal subset of CTCs (EMT-transformed) [10]. In prostate cancer it is especially important because the androgen-independent/castration-resistant form is highly aggressive and is associated with 5-year survival of less than 30% [13].

The lack of a specific detection marker for mesenchymal CTCs in epithelial cancers has hampered efforts to quantify these aggressive and metastatic cells. Recently, our laboratory demonstrated that vimentin, a member of the intermediate filament family of proteins and a marker of EMT [14], is expressed on the cell surface of sarcoma cells [15]. Since sarcoma cells are of mesenchymal origin and share a common phenotype with EMT cells, we explored the possibility of detecting EMT-like CTCs using 84-1 mAb against cell-surface vimentin (CSV) in PCA patients. Since other studies have also shown that EMT plays a critical role in PCA progression [16], the detection of CSV-CTCs is key to better understanding the etiology of PCA. In this study, we detected and enumerated CTCs from PCA patients using the CSV and CellSearch methods. We then compared the CTC counts from both methods with respect to clinical outcome and hormone sensitivity at the time of blood collection.

## RESULTS

### Detection of CSV-CTCs in PCA patients

**Figure 1:**
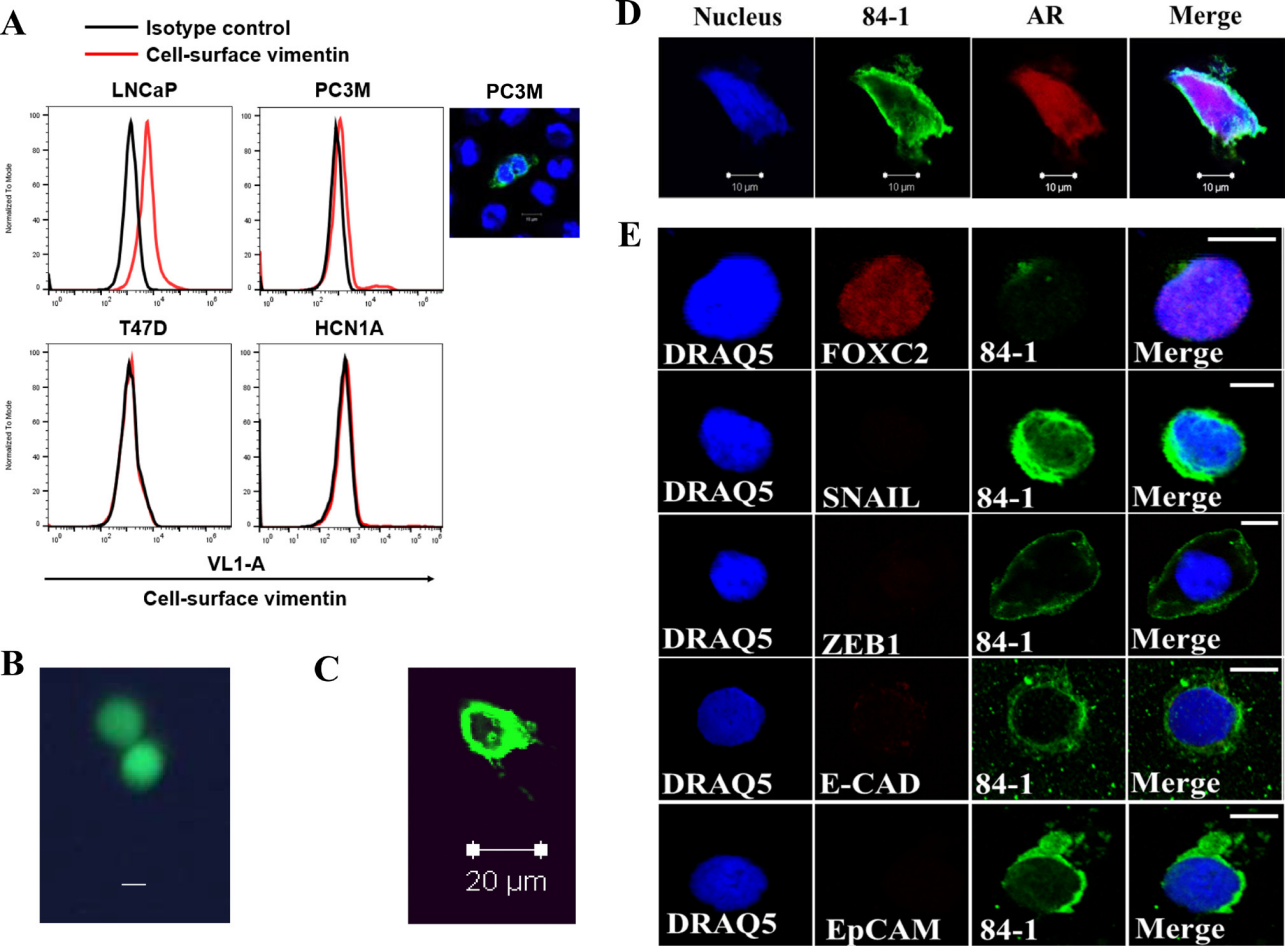
Detection of CSV-CTCs from patients with metastatic prostate cancer (**A**) Immunological assessment of CSV expression in CSV-expressing LNCaP, PC3M (cell staining shown adjacently), and CSV-nul T47D and HCN1A cell lines using flow cytometry. CSV was detected by 84-1 antibody (red line). Isotype control was used as negative control (black line). (**B**, **C**) Spiked Calcein AM stained LNCaP cells were isolated using CSV method and analyzed under fluorescent microscope, Scale bar, 10 μm (B) and confocal microscope (C). (**D**) CTC isolated from patient blood was stained for androgen receptor (AR) and CSV using 84–1. Scale bar, 10 μm. (**E**) CTCs stained with CSV and various markers as indicated. All experiments were done in triplicates.

We previously reported a method for isolating mesenchymal CTCs from sarcoma and CSV-CTCs from colon cancer patient blood samples [15, 17] and characterized the method in detail describing the sensitivity and specificity of CTC isolation along with validation studies. To determine whether PCA express CSV, we tested LNCaP, PC3M, T47D and HCN1A cells using flow cytometry, and the results indicated that these cells do express vimentin on their cell surface as indicated by the shift in the histogram of cells stained with 84-1 when compared to isotype control (Figure 1A). T47D and HCN1A cells are presented as a negative control. Also, only a partial number of cells are positive for CSV which can be attributed to the heterogeneity of cancer cells.

To evaluate if CSV is a good target to detect CTCs from blood of cancer patients, we first performed spiking assay, wherein LNCaP cells stained with Calcein AM were spiked into 7.5 mL blood and CSV positive cell capture method as previously reported was utilized to isolate the spiked cells (Supplementary Figure 1) [18]. The results show that the spiked cells were isolated and detectable (green) by fluorescence microscopy before fixation (Figure 1B) and after fixation under confocal microscopy (Figure 1C). Later, PCA patient derived blood specimens were tested for CTC detection using CSV positive cell capture method and the results indicated that the cells detected were positive for androgen receptor staining (Figure 1D) thus validating the prostate cancer identity of the CTC. As a negative control, we stained SHEP cells with the same anitbodies and found no AR staining (Supplementary Figure 2).

Furthermore, using CSV targeting 84-1 antibody, we detected CTCs from patients with metastatic PCA. CTCs from 10 patients were then isolated and characterized for their EMT phenotype using EMT-specific markers that included ZEB1, FOXC2, Twist, Snail, and Slug and the epithelial markers EpCAM and E-Cadherin. We were only able to detect FOXC2 in these cells (Figure 1E), while other EMT markers including Twist and Slug were nearly undetectable. However, the epithelial markers EpCAM and E-cadherin were absent in these cells, indicating a mesenchymal phenotype.

### Comparison of CTC quantification via CSV and CellSearch methods

**Figure 2:**
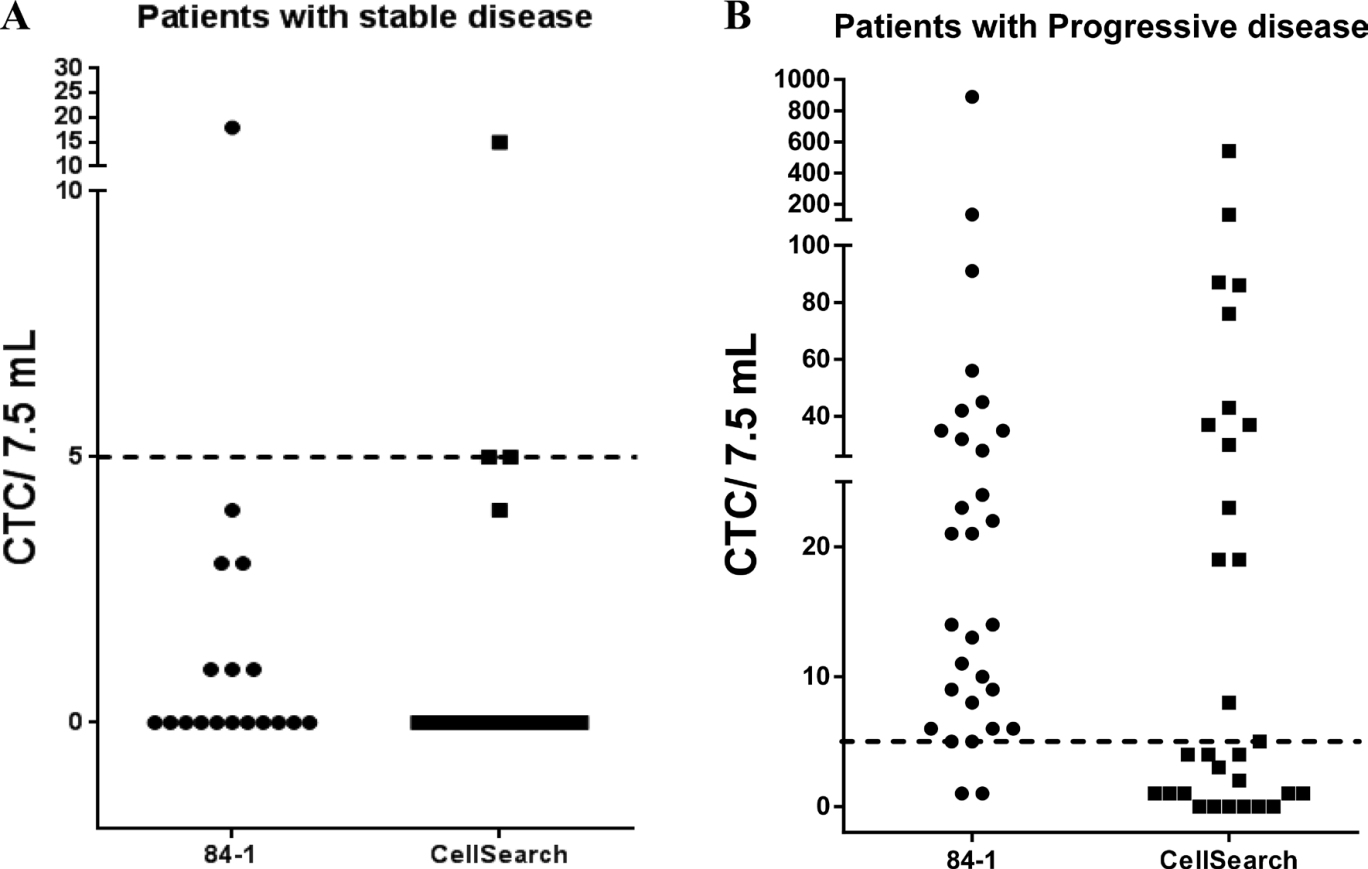
Quantification of CTCs from 48 patients with metastatic prostate cancer using the CSV and CellSearch method Two blood samples from each patient were obtained and analyzed using both the methods. Patients were classified as having progressive or stable disease based on clinical evaluations. CTC counts were plotted per 7.5 mL of blood. Dashed line indicates a threshold of 5CTCs/7.5 mL. (**A**) CTC enumeration in patients with responding/stable disease using both methods. (**B**) CTC enumeration in patients with non-responding/progressive disease using both methods. The data show that the CSV method is able to distinguish the progressive disease population with a higher sensitivity and specificity than that of the CellSearch method.

For this study, blood samples from a total of 48 patients with metastatic PCA (see details in Supplementary Table 1) were collected and analyzed using both the CSV and CellSearch methods. Also, 12 healthy patient blood samples were analyzed to determine the specificity of the methods, and no CTCs were detectable in any of these samples. Since this was a pioneer study analyzing CSV-CTCs from patients with PCA, we focused on isolating and quantifying CTCs from different patients with advanced stage PCA and those who were undergoing therapy at different stages (Table 1). All patients were classified as having responding/stable disease or non-responding/disease progression at the time of blood collection. This categorization was performed by the clinician to validate the role of CTCs in predicting therapeutic response in patients. Two blood samples were collected from each patient and each sample was analyzed using both methods. Since CellSearch uses a cut-off threshold of 5 CTCs/ 7.5 mL, we used the same threshold in our comparative analysis. Figure 2 depicts the quantification of CTCs from patients with stable disease (Figure 2A) and patients with progressive disease (Figure 2B) using both the CSV and CellSearch methods. The results, as presented in Table 2, showed that using the CSV method, we were able to isolate and quantify CTCs from patient blood samples and these results correlated with patients' disease status with very high sensitivity (93.33%) and specificity (94.4%). In comparison, using the same samples, the CellSearch method detected CTCs from patients with a sensitivity of 53.33% and with a specificity of 83.33% using 5 CTCs/7.5 ml cut off (Table 2); reducing cut off cell number reduced specificity for both methods. These results thus show that the CSV method was able to specifically and sensitively isolate CTCs from patients with progressive disease compared to that of CellSearch method.

**Table 1:** Characteristics of the patient population

Patients	Hormone Sensitive	Castration Resistant
*n* = 48	10 (20.83%)	38 (79.17%)
Gleason 6–7 (3 + 4)	3 (30.00%)	3 (7.89%)
Gleason 7 (4 + 3)–9	6 (60.00%)	26 (68.42%)
84-1 CTC Positive	6	31
CellSearch CTC Positive	5	24
**Met Sites**	**Hormone Sensitive**	**Castration Resistant**
Bone	8	35
Lymph nodes	5	20
Liver	2	4
Lung	1	2
**Age**	**Hormone Sensitive**	**Castration Resistant**
Mean	69.1	65.92
Median	69.5	67
Range	62–76	49–83
**PSA**	**Hormone Sensitive**	**Castration Resistant**
Mean	20.5	128.46
Median	9.15	24.25
Range	0.1–97.3	0.6–1442.2

**Table 2:** Diagnostic values of CTC count from both methods at selected CTC cutoff points

Biomarker	Cutoff point	Sensitivity	Specificity	Likelihood ratio
CTC counts 84-1 method	> 1	100%	61.1%	2.571
	> 2	77.93%	88.89%	8.4
	> 5	93.33%	94.4%	16.8
**CTC counts CellSearch method**	**> 1**	**80%**	**77.78%**	**3.6**
	**> 2**	**60%**	**77.78%**	**2.7**
	**> 5**	**46.67%**	**83.33%**	**2.8**

### ROC curves for CTC counts using both methods

**Figure 3:**
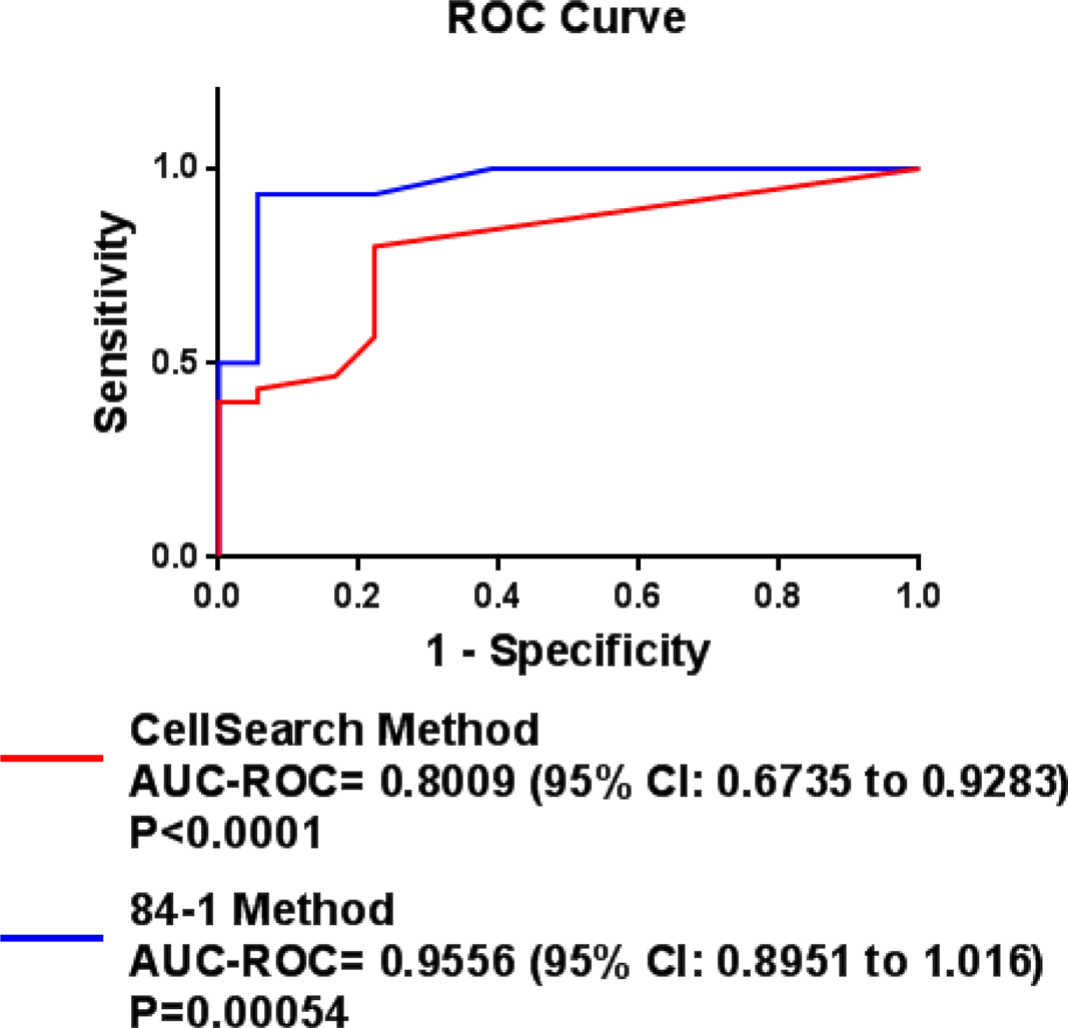
ROC curves for CTC counts using the CSV and CellSearch methods and serum PSA levels: ROC curves were determined to discriminate the patients with responding/stable disease from the patients with non-responding/progressive metastatic prostate cancer An AUC-ROC value of closer to 1 denotes a perfect method for discrimination.

ROC curves for CTC counts using the CSV and CellSearch methods were plotted to discriminate the progressing patients from the responding population. The ROC curves are depicted in Figure 3. Value closer to the AUC-ROC of 1 indicates greater discrimination of the given method. The AUC-ROC for the CSV method of CTC quantification in the discrimination between patients with stable and progressive disease was 0.9556 (*P* = 0.00054). A cutoff point of 5 CTCs/7.5 mL had a sensitivity and specificity of 93.3% and 94.4%, respectively. CTC counts from the CellSearch method had an AUC-ROC of 0.8009 (*P* < 0.0001), and a cutoff point of 5 CTCs/7.5 mL had a sensitivity and specificity of 83.33% and 33.33%, respectively. These results thus show that the CSV method has a greater sensitivity and specificity in discerning between populations with progressive vs stable disease.

### Higher CSV enriched CTC counts correlate with castration-resistant status of PCA

**Figure 4:**
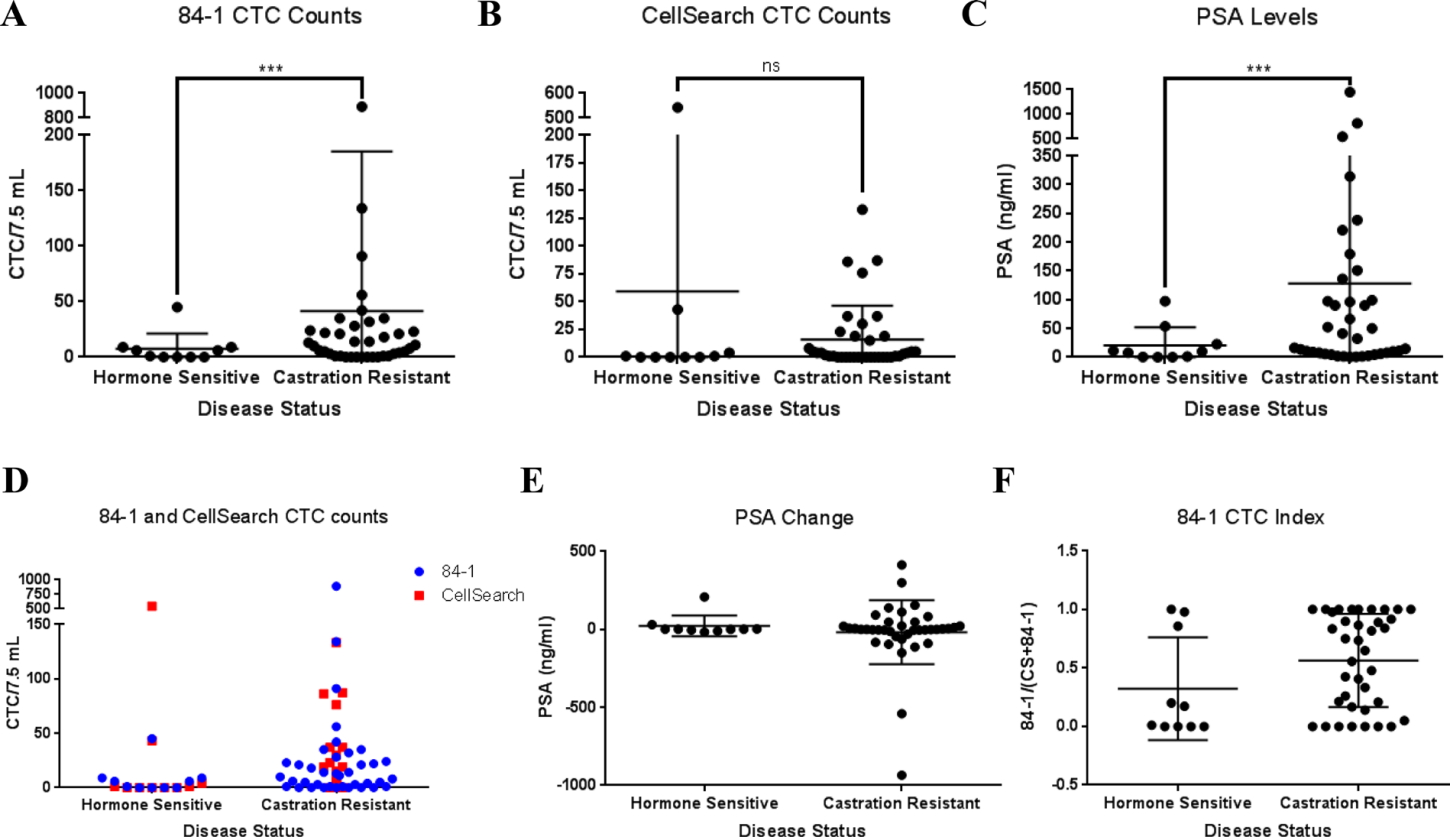
CSV CTC counts and PSA levels independently associate with castration resistance: Both CSV CTC counts (**A**) and PSA levels (C) but not CellSearch (**B**) are independent, and unrelated biomarkers of castration resistance in peripheral blood samples. Generalized poisson mixed regression was applied for CTC counts resulting in a *P* < 0.001 for (A) and *P* = 0.852 for (B). For PSA score, generalized log-normal mixed regression was applied which showed a similar *P* < 0.001 (**C**). CSV CTC count was significantly correlated to CellSearch CTC count (*P* = 0.003) using Spearman's rank correlation due to the non-normality of the data (**D**). Patient numbers for hormone sensitive and castration resistant groups are *n* = 10 and 38, respectively. PSA change (**E**) from time of liquid biopsy acquisition and 6 months afterwards did not show a significant difference between the two groups. Calculation of a CTC index as determined by the division of a patients CSV CTC count by the combined count of CSV and CellSearch did not yield a statistically significant difference (**F**).

The key driver of PCA is androgen signaling and as such, the primary means of inhibiting initial PCA growth is androgen ablation therapy. This includes physical or chemical castration and causes tumor regression until PCA adapts and becomes highly aggressive castration resistant type. To determine the relevance of CTC counts with hormone sensitivity, we segregated 48 PCA patient data into hormone sensitive and castration resistant groups (Table 1). Among our discoveries, CSV but not CellSearch CTC count was significantly correlated to castrate resistance, with higher CSV CTC counts for castrate-resistant group (*P* < 0.001 and *P* = 0.852, respectively) (Figure 4A, 4B). Similar correlation was also found in overall PSA score, with higher score for castrate-resistant group (*P* < 0.001, Figure 4C). We also found that CSV CTC count was significantly correlated to CellSearch CTC count (*P* = 0.003, Figure 4D). However, there was no correlation between PSA change and castrate resistance (*P* = 0.203, Figure 4E). We also analyzed the EMT CTC index relative to total CTC counts by dividing the number of CSV+ CTCs with the sum of CTCs obtained via either method for each patient. While average index value was higher for castration resistant patients, the overall difference between the groups was not significant (Figure 4F). On the whole, these data indicate that CSV may be instrumental in helping predict PCA progression towards hormone resistance. Our work shows for the first time that CSV-CTCs can be detected in PCA liquid biopsies and their higher counts be associated with the more aggressive, castration-resistance phenotype.

## DISCUSSION

There is an increasing need for the identification of novel specific and sensitive biomarkers that can predict the treatment response in PCA patients. Although serum PSA levels are routinely used as a biomarker for detecting and monitoring metastatic PCA, recent studies have revealed many shortfalls that render these levels unreliable [19]. As a result, alternative methods such as the detection of CTCs in patients with PCA has been explored.

The movement of cells from the primary tumor site to distant sites involves a complex mechanism(s) not yet fully understood. One of the cancer cellular programs involved in providing tumor cells with increased invasive and migratory capabilities is EMT, in which cells' phenotype is modified [12]. Recent evidence provides support for the notion that the migration and invasion potential of EMT-transformed CTCs is greater than that of their epithelial counterparts [12]. Moreover, cells that have undergone EMT demonstrate chemotherapy resistance and immune escape capabilities, providing these cells with greater odds of colonization. The EMT process thus plays a vital role in establishing a metastatic niche; it is critical to disease management that we are able to effectively detect and analyze these CTCs. Accordingly, it is essential to identify a novel biomarker with high sensitivity and specificity that can accurately detect EMT-transformed CTCs from the blood of cancer patients. Furthermore, enumeration of these CTCs may hold clinical relevance towards castration resistance status. To address this gap in knowledge, we evaluated CSV as a novel CTC marker. In previous work [14] we showed that CSV is capable of detecting mesenchymal CTCs from sarcoma patients and EMT CTC in colon cancer [17]; in this study, we demonstrated for the first time in our knowledge the isolation, detection, and quantification of EMT-transformed CTCs in patients with PCA.

In PCA, the implementation of techniques capable of detecting EMT-transformed CTCs is lacking. To the best of our knowledge, all PCA CTC research has been published regarding their counts as prognostic indicators of overall survival and/or therapeutic resistance [20, 21]. No research has yet produced the utility of CTC counts as indicators of hormone responsiveness. This may be the result of near-universal reliance of PCA CTC detection on the epithelial marker EpCAM-based CellSearch technology. Our EMT-CTC profiling (Figure 1E) data show that the CTCs are heterogeneous in nature and under constant influence of the harsh environment in the blood that might alter their gene expression profile. It is interesting to note that the majority of researchers have observed an irregularity in EMT marker expression in PCA cells [16]. It is possible that these CSV-CTCs mainly harbor FOXC2 expression, which has been characterized in a large number of patient populations.

Our analysis suggest that EMT-CTC have a better prognostic role in castration resistance than EpCAM CTC (Figure 4A, 4B). It is possible that some of the CTCs isolated by CellSearch may also be expressing CSV, which would imply at least some overlap in the CTCs captured by the two methods [22]. However this aspect needs to be further validated. Meanwhile, it is worth exploring if a combination of EMT-CTC and PSA can provide better prognosis of castration resistance. Since it has been shown that androgen receptor (AR) mutations can be detected in PCA CTCs [23], future research using our method may yield greater information regarding AR status in CSV-CTCs. Such knowledge can significantly improve the early detection of the onset of androgen independence.

Recently, another group has retrospectively analyzed vimentin expression in CTCs collected by CellSearch which are most likely epithelial due to the use of EpCAM-reliant method [22]. Here, vimentin was found as a stronger indicator of OS than Ki67. However, ours is the first study to compare EMT-like CTCs (isolated via the CSV method) with epithelial CTCs (isolated using the CellSearch method), and serum PSA levels to discriminate the population with disease progression from the population with treatment response. Our data demonstrate that CSV is a highly sensitive and specific marker that may assist in assessing treatment efficacy and the onset of castration resistance in patients with PCA; however, an independent prospective study with a large population will be necessary to fully validate the efficiency of this method. Also in parallel with a longer follow-up of patients will reveal the clinical significance of CTCs in predicting survival, relapse, or the effect of changing treatment.

## MATERIALS AND METHODS

### Patients and study design

This study was approved by The University of Texas MD Anderson Cancer Center Institutional Review Board. Peripheral blood samples from 48 metastatic PCA patients were collected for this study. These patients were diagnosed and treated for metastatic PCA. Clinico-pathological information was recorded for all patients at the time of blood collection. RECIST guidelines were used to evaluate the disease status of the patient (responding/ stable and non-responding/ progression). Also, blood from 12 healthy volunteers (ages 18-60 years) was collected to assess the specificity of CTC detection using 84-1 antibody method. All patients bear relapsed or advanced PCA and blood was collected at regular clinical checkup. Based on clinical evaluation at the time of blood collection using the RECISTv1.1 criteria, the patients were classified as responding/stable disease or non-responding/disease progression. For each patient, 7.5 mL blood was collected in each of two CellSave collection tubes (Janssen Diagnostics LLC, NJ, USA) for the CellSearch test. One blood sample was used to quantify CTCs using CellSearch and the other sample was used to quantify CTCs using 84-1 monoclonal antibody.

### Flow cytometry

A total of 5 × 10^5^ LNCaP, PC3M, and T47D cells were detached with a non-enzymatic dissociation buffer, washed, and stained for 20 min on ice in the dark. For CSV analysis, cells were stained with the 84-1 monoclonal antibody we developed (1:100) [15]; mouse primary antibody (Invitrogen) was used as an isotype control. Later, cells were rinsed twice in phosphate-buffered saline (PBS) and labeled for secondary antibody using Alexa Fluor-405 secondary antibody (Invitrogen). Cells were then washed twice in PBS and immediately used for data acquisition using an Attune flow cytometer (Applied Biosystems). For analysis, 50,000 cells were counted and plotted as a histogram using VL1 (off 405 nm laser) - Filter (Center/Bypass): 450/40 nm. The data were analyzed using FlowJo software (Treestar).

### Quantification of CTCs via cell search

CTCs were quantified via CellSearch according to the manufacturer's protocol and training. Detailed methods of quantification and cell counting by a pathologist have been described elsewhere [10].

### Quantification of CTCs via 84-1 antibody

CTCs were quantified via 84-1 antibody as previously reported [10, 17, 18]. Briefly, after mono-nucleated cell isolation using BD Vacutainer CPT cell preparation tubes, CD45 positive cells were depleted using EasySep Human CD45 Depletion Kit (Stem Cell Technologies) according to the manufacturer's recommendations. To minimize nonspecific binding, antibody against human Fc receptor was added to the cocktail. Second, the CD45 negative cell fraction was subjected to 84-1 positive selection. Cells were labeled with 84-1 antibody, and later mouse immunoglobulin G binding microbeads (Miltenyi Biotec) were added to the mixture. Then, 84-1 positive cells were extracted using a magnetic column (Miltenyi Biotec) according to the manufacturer's recommendation. The cells thus obtained were 84-1 positive or CD45 negative and were further validated using immunostaining for 84-1 (species: mouse) and CD45 (species: rabbit); later, images were obtained using confocal microscopy. To validate the tumor specific origin of CTC isolated from patients' blood, the CTCs were stained for Androgen receptor (D6F11) XP^®^ Rabbit mAb #5153, cell signaling). Cells were then rinsed in PBS (pH 7.4) and stained with Alexa Fluor-555 secondary antibodies (Invitrogen) (1:250) for EpCAM (species: rabbit) and Alexa Fluor-647 for 84-1 (species: mouse). For nuclei staining, Sytox Green (Invitrogen) (1:1000) was incorporated along with secondary antibody for 60 min. The cells were then washed with PBS (pH 7.4) three times for 15 min each and mounted in SlowFade antifade reagent (Invitrogen). For confocal analysis, images were acquired in 8 bits with the Zeiss LSM 510 confocal microscope using LSM5 image capture and analysis software, version 3.2 (Zeiss). A 63x water-immersion objective lens (NA, 1.0) was used with digital zoom for image capture. All images were acquired by the same operator using the same intensity and photo detector gain in order to allow quantitative comparisons of relative levels of immunoreactivity between different samples. Two independent researchers were involved in the detection and quantification of CTCs to prevent any user variability. In samples wherein there were amounts of blood other than 7.5 mL, the data were normalized to express the CTC counts in 7.5 mL.

### Spiking assay

To demonstrate the capturing precision and reproducibility by 84-1 antibody, cultured LNCaP cells were spiked in to blood collected from healthy patient. ∼5 LNCaP cells were spiked in to 7.5 mL of blood. After isolation of cells using 84-1 method, the cells were fixed using 4% paraformaldehyde, stained with 84-1 and CD45 and visualized under confocal microscope (Zeiss LSM510).

### Statistical methods

Analysis of two groups of data for the differences between the means was determined using a Student *t* test. Correlations and outcomes of agreement between both the methods were assessed by κ-test. In all cases, a *P* value of < 0.05 was considered statistically significant. Diagnostic performance of CTC quantification using either of the methods and serum PSA levels was assessed by constructing a receiver operating characteristic (ROC) curve and was further evaluated by calculating the area under each ROC curve (AUC-ROC) [24]. An AUC-ROC value of 1 denoted that the test method was able to discriminate perfectly, whereas an AUC-ROC value of 0.5 denoted a worst discrimination of the test. A *P* value was calculated for the difference between each AUC-ROC.

The correlations between the right-skewed 84-1 CTC counts, CellSearch CTC counts and PSA scores in PCA patient data were examined using exact test for Spearman's rank correlation coefficient. To compare the CTC count difference between castrate-sensitive and castrate-resistant groups, we applied the mixed-effects Poisson regression when tumor type was treated as random effects. For right-skewed continuous variable PSA score, mixed-effects log-linear regression was applied when tumor type was treated as random effects for comparison between castrate -sensitive and castrate-resistant groups.

Data analysis was conducted using GraphPad-Prism v-5.0 (La Jolla, CA) and R v3.1.2 with packages lme4 v1.1-7. *P* values less than or equal to 0.05 were considered statistically significant.

## SUPPLEMENTARY MATERIALS FIGURES AND TABLE




