# Supplementary material for: EMT circulating tumor cells detected by cell-surface vimentin are associated with prostate cancer progression
Source: Oncotarget. 2017 May 4;8(30):49329–37. doi: 10.18632/oncotarget.17632 (PMC5564771; doi:10.18632/oncotarget.17632)
Supplement: Supplementary file 1 [file oncotarget-08-49329-s001.pdf]

EMT circulating tumor cells detected by cell-surface vimentin are associated with prostate cancer progression

SUPPLEMENTARY MATERIALS

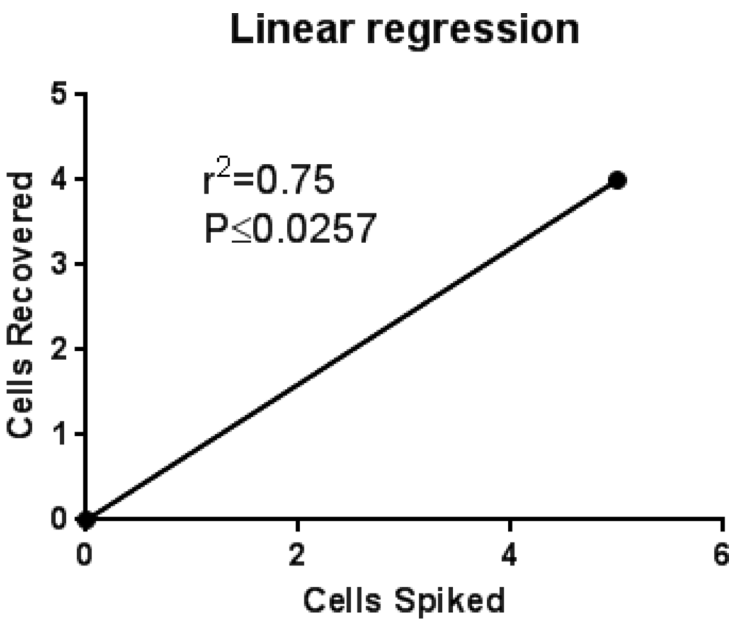

Supplementary Figure 1: LNCaP cell spiking assay. Linear regression plot of ~5 LNCaP cells spiked into healthy donor human blood and the rate of recovery.

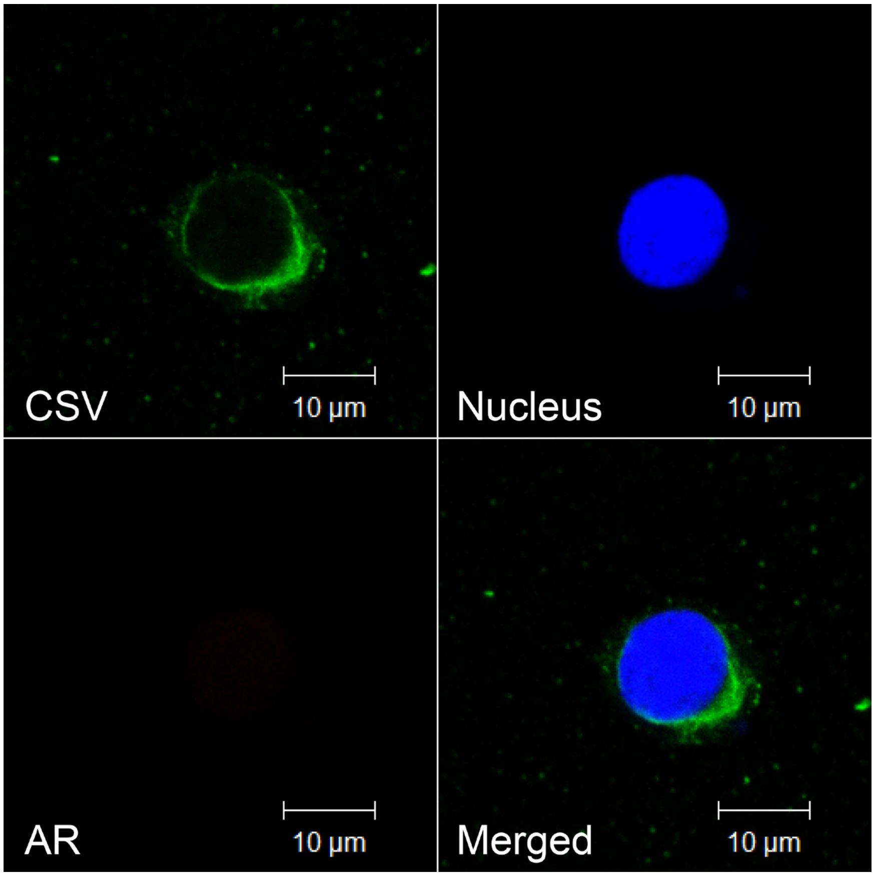

Supplementary Figure 2: SHEP cell stained for CSV and AR. Representative image of an SHEP cell stained with CSV and AR as indicated.

Supplementary Table 1: Clinical parameters for all metastatic prostate cancer patients. See Supplementary_Table_1.
